# Rubber Attachment

**Published:** 1863-06

**Authors:** 


### RUBBER ATTACHMENT.

A few days ago we saw a piece of work from the office of Dr. Moore, of Lafayette, Ind., which, for style and neatness of finish, we have seldom seen equalled. The teeth are in sections and carved for the case, and attached to gold plate with rubber. The gum on the palatine surfaces of the teeth extended to and fitted upon the plate very closely. There are grooves or holes in the base of the sections, so arranged that when filled with vulcanized rubber, the teeth are held firmly; and upon the plate immediately under the grooves are soldered loops which hold the rubber firmly. When the work is finished, no rubber is seen, and yet the attachment is very strong. The exact method of putting up the work we are not now competent to give; but hope Dr. Moore will favor us with an exact description of the process at his earliest covenience. T.

